# Hyponatremia Associated with Heart Failure: Pathological Role of Vasopressin-Dependent Impaired Water Excretion

**DOI:** 10.3390/jcm4050933

**Published:** 2015-05-08

**Authors:** San-e Ishikawa

**Affiliations:** Department of Medicine, Saitama Medical Center, Jichi Medical University, 1-847 Amanuma Omiya, Saitama 330-8503, Japan; E-Mail: saneiskw@jichi.ac.jp; Tel.: +81-48-647-2111; Fax: +81-48-648-5166

**Keywords:** hyponatremia, arginine vasopressin (AVP), aquaporin2 (AQP2), non-osmotic release, heart failure, cardiac output, circulatory blood volume, impaired water excretion, AVP V_2_ receptor antagonist

## Abstract

An exaggerated increase in circulatory blood volume is linked to congestive heart failure. Despite this increase, reduction of the “effective circulatory blood volume” in congestive heart failure is associated with decreased cardiac output, and can weaken the sensitivity of baroreceptors. Thereafter, tonic inhibition of the baroreceptor-mediated afferent pathway of vagal nerves is removed, providing an increase in non-osmotic release of arginine vasopressin (AVP). In the renal collecting duct, the aquaporin-2 (AQP2) water channel is regulated by sustained elevation of AVP release, and this leads to augmented hydroosmotic action of AVP, that results in exaggerated water retention and dilutional hyponatremia. Hyponatremia is also a predictor for worsening heart failure in patients with known/new onset heart failure. Therefore, such a dilutional hyponatremia associated with organ damage is predictive of the short- and long-term outcome of heart failure.

## 1. Introduction

An exaggerated increase in circulatory plasma volume is linked to organ disorders such as congestive heart failure, decompensated liver cirrhosis and nephrotic syndrome, in which non-osmotic release of arginine vasopressin (AVP) is associated with an increase in water permeability in cells of the renal collecting duct [1,2]. Sustained AVP-dependent antidiuresis produces water retention, thus increasing the circulatory blood volume in congestive heart failure. An excessive circulatory plasma volume results in dilutional (hypervolemic) hyponatremia [3]. The pathogenesis of impaired water excretion in congestive heart failure is examined in this review. First, the physiological control of AVP release, and inappropriate release of AVP in heart failure are described. Second, the augmented hydroosmotic action of AVP and expression of aquaporin-2 (AQP2) water channel are discussed in association with water retention. Finally, the ability of organ damage-related dilutional hyponatremia to predict long-term outcome in heart failure is examined.

## 2. Osmotic and Non-Osmotic Control of AVP Release

AVP is a peptide hormone containing nine amino acids, that is synthesized in both magnocellular and parvocellular neurosecretory neurons of the hypothalamus. The major projection of magnocellular neurosecretory neurons is to the posterior pituitary gland. The magnocellular neurosecretory neurons in the supraoptic and paraventricular neurons respond to both osmotic and non-osmotic stimulation, and are involved in control of body water content and blood pressure. The axon terminals of paraventricular neurons reach the median eminence of the hypothalamus and certain areas of the central nervous system. AVP stimulates ACTH release and also acts as a neurotransmitter with potential roles in a variety of functions such as social behavior, emotional response and others [4].

**Figure 1:**
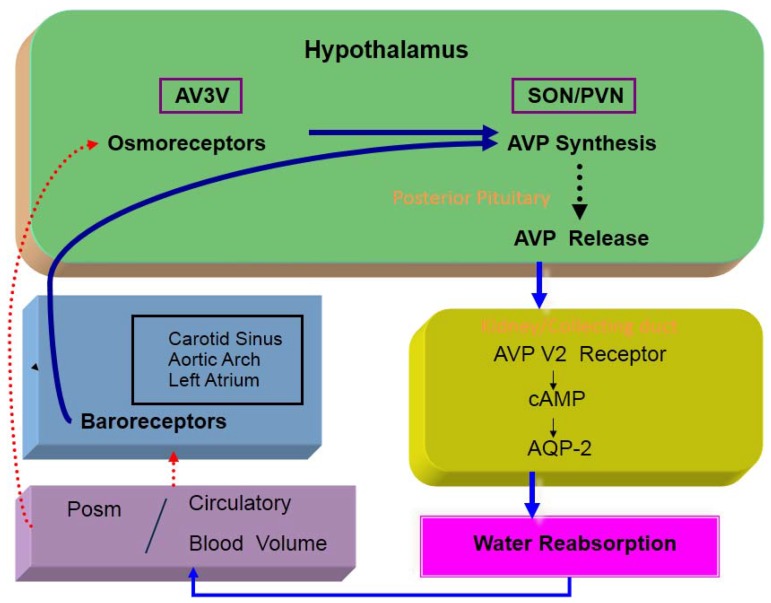
Osmotic and non-osmotic control of AVP release.

Osmotic and non-osmotic stimulations are the two major factors that control AVP release [5] (Figure 1). Osmoreceptors reside in the anteroventral third ventricle (AV3V) region of the hypothalamus, particularly in the organum vasculosum of the lamina terminalis (OVLT). Osmoreceptors are located outside the blood-brain barrier, and are very sensitive to changes in plasma osmolality (Posm). There are neural inputs from the osmoreceptors to the supraoptic and paraventricular nuclei. Afferent fibers from arterial baroreceptors terminate in the nucleus of the tractus solitaries (NTS) of the dorsomedial medulla oblongata [6]. A1 adrenergic neurons of the ventrolateral medulla are involved in the afferent pathway from the NTS to the neurosecretory AVP cells. Studies using interruption of the glossopharyngeal and vagal afferent pathways from baroreceptors have demonstrated the potency of non-osmotic AVP stimulation [7,8].

There is a close correlation between Posm and plasma AVP levels in healthy subjects and in subjects with various levels of hydration [5]. The osmotic threshold for AVP secretion is approximately 280 mmol/kg in healthy subjects [9]. Osmoreceptors are remarkably sensitive, since a 1-mmol/kg change in Posm could alter AVP release; however, the sensitivity is influenced by the nature of solutes, the rate of change in Posm, age and alcohol intake [10].

Decreases in arterial blood pressure and circulatory blood volume diminish the sensitivity of high-pressure and low-pressure (left atrial) baroreceptors, and are potent non-osmotic stimuli for AVP release. The baroreceptor-mediated afferent pathway for AVP release is also activated by several factors, including low cardiac output, left atrial distension, atrial tachycardia and hypoxia [5].

It is of value to recognize the independence of osmotic and non-osmotic control of AVP release. An electrophysiological study verified that the osmotic and non-osmotic pathways independently enter the same AVP neurons of the paraventricular and supraoptic nuclei [11]. Many clinical osmolar disorders can be interpreted based on competitive inputs of baroreceptor and osmoreceptor pathways into the same population of neurosecretory cells, independent of an intrinsic alteration in osmoreceptor sensitivity.

## 3. Hydroosmotic Action of AVP and Aquaporin2 Water Channel

### 3.1. Hydroosmotic Action of AVP

**Figure 2:**
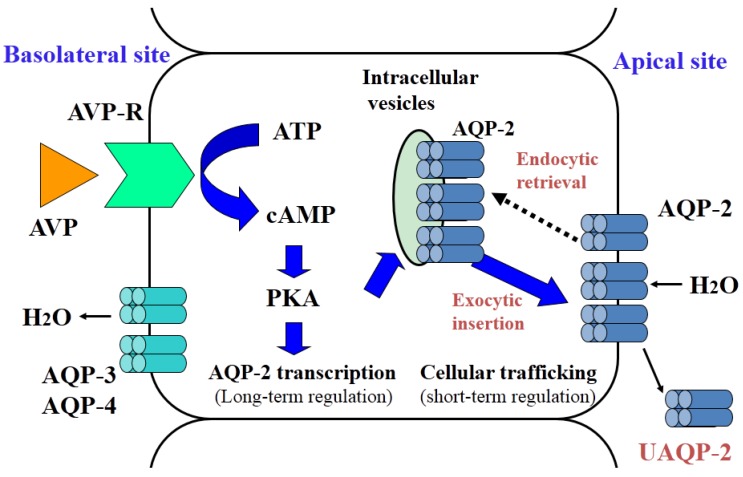
Cellular action of AVP in renal collecting duct cells.

AVP V_2_ receptors on the basolateral membranes of renal collecting duct cells are functionally coupled to the Gs proteins, leading to the activation of adenylate cyclase [12]. The human V_2_ receptor cDNA encodes 371 amino acids and has seven transmembrane domains, which is characteristic of G-protein-coupled receptors [13]. Receptor occupancy with AVP leads to a conformational change in the receptor and subsequent replacement of guanine diphosphate with guanine triphosphate in the Gs α-subunit. This allows the activation of adenylate cyclase to produce cAMP, which activates protein kinase A (PKA) and is catabolized by cAMP-dependent phosphodiesterase. Phosphorylation of PKA then mediates cellular signaling of AVP to the aquaporin2 (AQP2) water channel. This leads to translocation of AQP2 from the membranes of cytoplasmic vesicles to the apical plasma membrane and an increase in AQP2 expression (Figure 2).

### 3.2. Aquaporin 2: Structure and Function

Sasaki and his group [14,15] cloned the cDNA of rat and human AQP2, both of which contain 271 amino acids and have six putative transmembrane domains, an internal tandem repeat and a conserved asparagine-proline-alanine (NPA) box. The *AQP2* gene maps to chromosome 12q13. Northern blot analysis showed that AQP2 mRNA is expressed in the kidney, and immunocytochemistry analysis revealed that AQP2 is localized only in the principal cells of the renal collecting duct. AQP2 staining is strong in the apical and subapical regions. Immunoelectron micrcoscopy showed that AQP2 resides in cytoplasmic vesicles in the subapical region in a normally hydrated condition. Administration of exogenous AVP or dehydration causes translocation of AQP2 from the cytoplasmic vesicles to the apical plasma membrane.

AVP is responsible for short-term and long-term regulation of AQP2. Short-term regulation by AVP involves cellular trafficking of AQP2 to the apical membrane of collecting duct cells [14]. AQP2 phosphorylation is required for AQP2 translocation to the apical membrane. AQP2 forms homotetramers, and at least three of the four monomers in an AQP2 tetramer must be phosphorylated for cellular trafficking. AVP-dependent protein kinase A activation is required to effectively phosphorylate AQP2, and this process is assisted by protein kinase A anchoring proteins. There are four phosphorylation sites near the AQP2 c-terminus. Among them serine 256 is the most important phosphorylation site of AQP2. Noda *et al.* [16,17] determined the details of the intra-complex transaction of AQP2 movement in the cells. Under basal conditions, AQP2 binds to G-actin, while F-actin is stabilized by tropomyosin 5b (TM5b) to form a barrier that inhibits translocation of AQP2 to the apical plasma membrane. AVP-triggered AQP2 phosphorylation releases AQP2 from G-actin and promotes AQP2 association with TM5b, which sequesters TM5b from F-actin and destabilizes the F-actin network, allowing efficient movement of AQP2 to the apical plasma membrane.

In addition to controlling cellular trafficking of AQP2, AVP regulates the total number of AQP2 water channels in collecting duct cells. AVP stimulates expression of AQP2 mRNA, followed by the synthesis of AQP2 protein [15,18]. There is no change in AQP2 mRNA and protein expression after water deprivation in homozygous Brattleboro rats, which have no endogenous AVP [19]. In response to a forced water load in 72-h dehydrated rats, the expression of AQP2 mRNA was profoundly reduced in parallel with normalization of plasma AVP levels [20]. A 50% reduction in AQP2 mRNA expression occurred within 30 min. Therefore, AVP plays a pivotal role in the on-off regulation of the cellular trafficking of AQP2 and the synthesis of AQP2 in collecting duct cells.

As mentioned above, non-suppressible AVP release is directly involved in abnormal antidiuresis in pathological states of impaired water excretion. Such a chronic excess of AVP is likely to be closely associated with the abundance of AQP2 protein in collecting duct cells despite hypoosmolality. Thus, long-term regulation of AQP2 may be a major factor in the impairment of water excretion.

## 4. Impaired Water Excretion in Congestive Heart Failure

### 4.1. Pathogenesis of AVP Secretion

In several animal models of low-output and high-output cardiac failure and in congestive heart failure in humans, it has been demonstrated that plasma AVP, renin activity, aldosterone and norepinephrine are significantly increased [21,22,23,24,25,26]. There was no controversy in augmented hormonal release among the studies.

**Figure 3:**
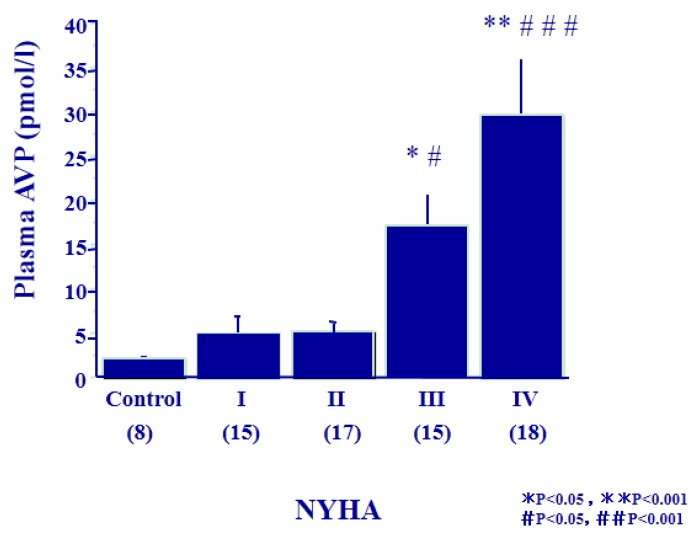
Distribution of Plasma AVP levels in the 65 patients with congestive heart failure. Cited from [27].

**Figure 4:**
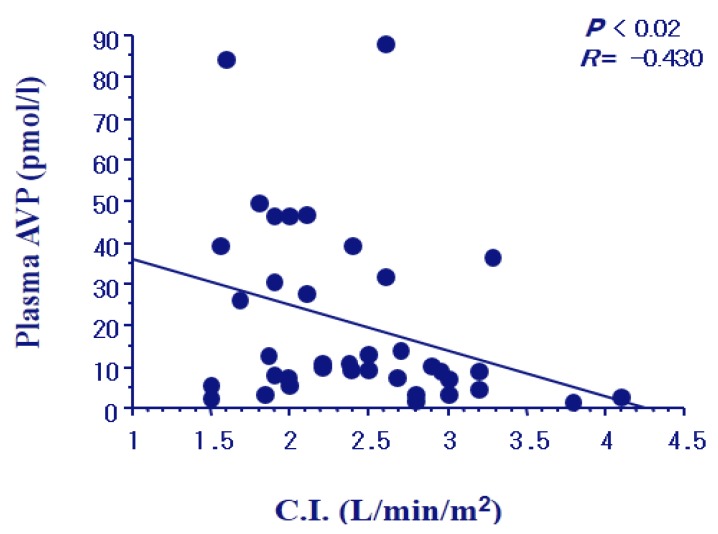
Negative correlation of plasma AVP levels with cardiac index (CI) in the patients with congestive heart failure.

We examined plasma AVP levels and urinary excretion of AQP2 in 65 patients with congestive heart failure [27]. The patients were 51 males and 14 females, with the ages of 62.0 ± 12.0 years. The patients included class I through class IV of New York Heart Association (NYHA) criteria. As shown in Figure 3, plasma AVP levels were increased gradually with an increase in the NYHA class in the patients. The levels were elevated to 17.2 and 29.4 pg/mL (mean) in the subgroups of NYHA class III and IV, respectively, compared to controls (* *p* < 0.05 and ** *p* < 0.001). Plasma AVP levels in NYHA III and IV were also significantly higher than that in NYHA class I (# *p* < 0.05 and ## *p* < 0.001). Swan-Ganz catheterization was carried out to determine the cardiac hemodynamics. Cardiac index gradually decreased according to the severity of NYHA class. Plasma AVP levels had a negative correlation with the cardiac index (*r* = −0.430, *p* < 0.02) (Figure 4). Elevation of AVP release was closely associated with the afferent pathway of baroreceptors, which was stimulated by the reduced “effective circulatory blood volume” as described below.

**Figure 5:**
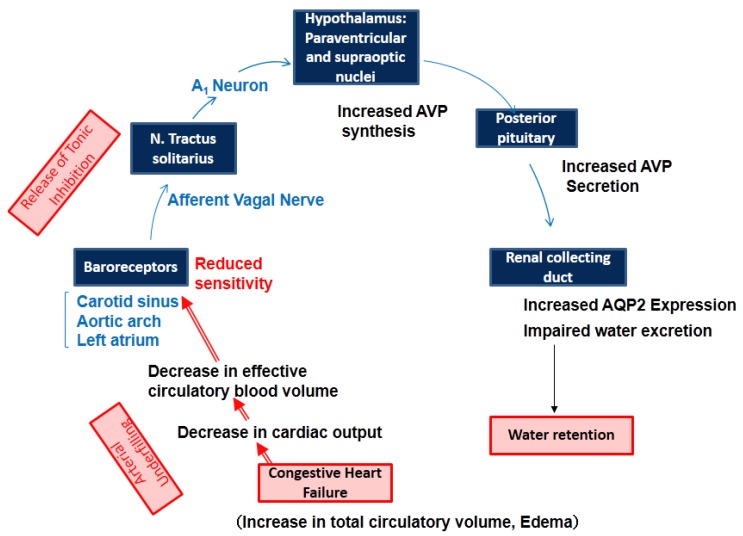
Pathological role of AVP in impaired water excretion in congestive heart failure.

Renal sodium and water excretion are predominantly regulated by the integrity of the arterial circulation, which is determined by the cardiac output and peripheral vascular resistance. Several baroreceptors on the high pressure side of the circulation can sense arterial underfilling. These receptors resided in the left atrium, carotid sinus, aortic arch and renal afferent arterioles. Reduction in baroreceptor sensitivity occurs due to a decrease in systemic arterial pressure, stroke volume, renal perfusion or peripheral vascular resistance [28]. In cardiac failure, cardiac output is decreased in association with reduced stroke volume despite an increase in total circulatory blood volume (Figure 5). We have proposed the term of “effective circulatory blood volume” to describe this condition. A decrease in effective circulatory blood volume impairs the sensitivity of baroreceptors, which leads to an increase in the activity of the sympathetic nervous system, the activation of the renin-angiotensin-aldosterone system and non-osmotic release of AVP. However, it remained unclear how baroreceptors sense the decrease in effective circulatory blood volume linked to low cardiac output in congestive heart failure.

### 4.2. Impaired Water Excretion

Several studies in animal models of impaired water excretion have shown that enhanced hydroosmotic action of AVP depends on non-suppressible release of AVP. Two groups of investigators have shown an increase in AQP2 protein abundance in rats with congestive heart failure. Xu *et al.* [29] produced chronic heart failure by ligating the left coronary artery. Cardiac output and plasma osmolality were significantly decreased and plasma AVP increased in the chronic heart failure rats compared to the sham-operated rats. AQP2 mRNA and protein abundance were both significantly increased in the kidneys of the rats with chronic heart failure. Similarly, Nielsen *et al.* [30] used immunocytochemistry and immunoelectron microscopy to show enhanced trafficking of AQP2 to the apical plasma membrane in collecting duct cells in heart failure rats. Administration of OPC-31260, an AVP V_2_ receptor antagonist, significantly diminished the AQP2 mRNA expression and protein abundance.

**Figure 6:**
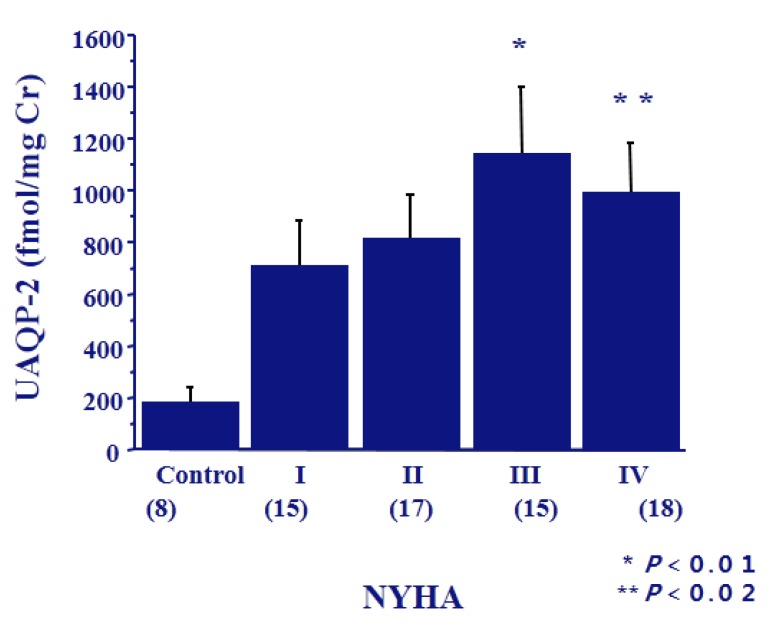
Urinary excretion of AQP2 in the patients with congestive heart failure. Cited from [27].

AQP2 has been detected in urine as both soluble and membrane-bound forms by western blot analysis [31]. Quantitative analysis of urinary excretion of AQP2 can be performed by radioimmunoassay or ELISA. Exogenous administration of AVP elicits a prompt increase in urinary excretion of AQP2 in normal subjects and in patients with central diabetes insipidus [31]. The fraction of AQP2 excreted into the urine is approximately 3% of the AQP2 present in collecting duct cells [32]. We found a positive correlation between urinary excretion of AQP2 and plasma AVP levels in humans [33]. Urinary excretion of AQP2 increased in the patients with congestive heart failure with higher NYHA class (Figure 6) [21], and also had a significant positive correlation with plasma AVP levels. The finding of exaggerated urinary excretion of AQP2 may be tightly linked with the upregulation of AQP2 mRNA expression in the kidneys. Similar results were obtained in other disorders of impaired water excretion, including syndrome of inappropriate secretion of antidiuretic hormone (SIADH), hypopituitarism and mineralocorticoid-responsive hyponatremia of the elderly (MRHE) [34]. Taken together, these findings suggest that elevated urinary excretion of AQP2 is caused by the augmented hydroosmotic action of AVP.

In addition to V_2_ receptors, there are V_1_ receptors in various tissues, including in vascular smooth muscle cells, glomerular mesangial cells, and liver cells. V_1b_ receptors are found in the pituitary gland. The post-receptor signal is mediated through phosphatidylinositol metabolism and cellular free calcium mobilization. Theoretically, elevated plasma AVP may act on V_1a_ receptors and mimic blood pressure control, but there is no clinical evidence for modulating blood pressure via V_1a_ receptors in heart failure patients.

As mentioned above, besides an increase in the AVP release baroreceptor-mediated afferent pathway of vagal nerves activates sympathetic nervous system. The sympathetic activation induces in the kidney arteriolar vasoconstriction, followed by both glomerular filtration rate reduction and proximal Na and water hyper-reabsorption, with a reduced distal delivery of water and Na. Also, stimulation of renal sympathetic nerves activates renin-angiotensin-aldosterone system responsible for distal Na and water hyper-reabsorption.

### 4.3. Hyponatremia in Congestive Heart Failure

**Figure 7:**
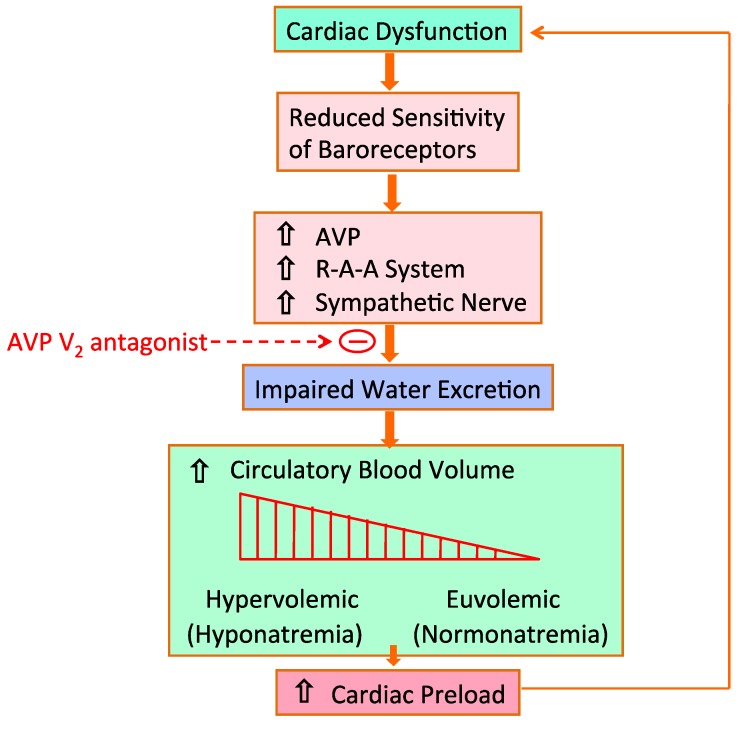
Baroreceptor-mediated hormone-dependent water retention in heart failure. Cited from [39].

Hyponatremia is a hallmark of congestive heart failure. Gheorghiade *et al.* [35] found hyponatremia of less than 135 mmol/L in 19.7% of 48,612 patients with congestive heart failure. Additional studies [36,37,38] have also shown hyponatremia in congestive heart failure. As aforementioned, exaggerated antidiuresis produces AVP-induced water retention, which results in an increased circulatory blood volume. Also, baroreceptor-mediated activation of the renin-angiotensin-aldosterone system and sympathetic outflow from the central nervous system increases sodium reabsorption in the kidney. However, compared to sodium retention, water retention is the predominant phenomenon in clinical manifestation of dilutional hyponatremia. Even patients with normal serum sodium have an underlying disorder of water retention in congestive heart failure. As shown in Figure 7, alteration of serum sodium is a successive phenomenon from euvolemic to hypervolemic state, and severe water retention results in dilutional (hypervolemic) hyponatremia. Excessive circulatory blood volume increases cardiac preload in the failing heart and further deteriorates impairment in cardiac contraction [39].

### 4.4. Hyponatremia Predicts Prognosis

As mentioned above, hyponatremia is relatively common in patients with congestive heart failure [35]. Hyponatremia was defined as a serum Na levels below 135 mmol/L and was shown to be predictive of short-term and long-term outcomes in patients. When serum Na levels were below 135 mmol/L, length of stay in hospital and in-hospital mortality were increased compared with those more than 135 mmol/L [35]. Also, long-term prognosis is related to the admission serum Na levels. Namely, lower serum Na levels at admission significantly related to increased post-discharge mortality and re-hospitalization due to recurrence of heart failure [35,36,37]. Thus, the admission serum Na levels is a significant independent predictor of in-hospital mortality, post-discharge mortality and re-hospitalization.

**Figure 8:**
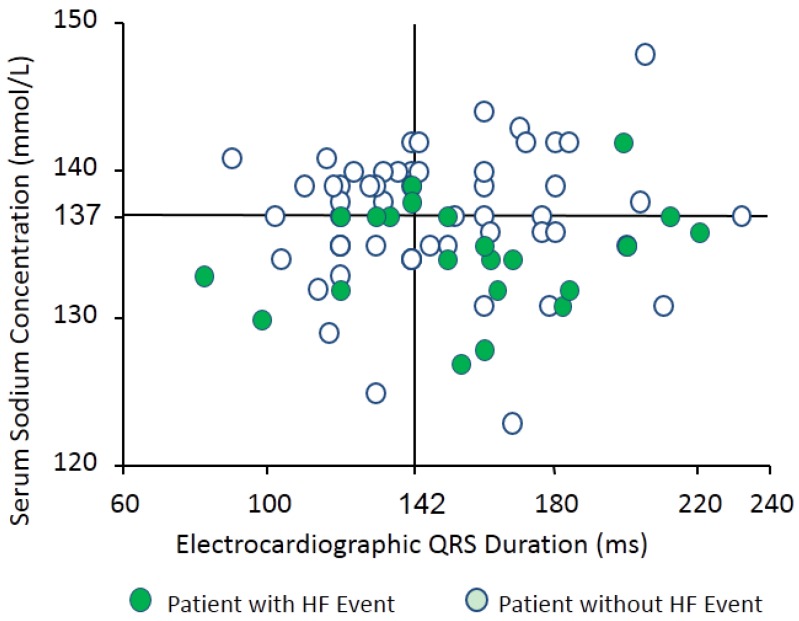
Distribution of serum sodium levels and electrocardiographic QRS duration in patients with congestive heart failure. Cited from [38].

We found that hyponatremia is also a predictor for worsening heart failure in patients receiving cardiac resynchronization therapy (CRT) [38]. Seventy-seven patients with chronic heart failure receiving CRT were enrolled. The patients were all in NYHA II, III or IV, and their left ventricular ejection fraction on echocardiogram was less than 35%. During a median follow-up period of 601 days, 22 of 77 patients (29%) had heart failure events. In multivariate analysis, a low serum Na concentration was the only factor associated with the occurrence of heart failure events (Hazard ratio 0.82, *p* = 0.034) among the parameters with a *p* value < 0.10 in the univariate analysis [38]. Admission serum Na levels had significant negative correlations with pulmonary capillary wedge pressure and with plasma AVP levels, thus indicating inappropriately the non-osmotic release of AVP in congestive heart failure [38]. Figure 8 shows the distribution of the parameters of admission electrocardiographic QRS duration and serum Na levels. A high incidence of heart failure was found in hyponatremic patients, especially those with widened QRS duration.

### 4.5. AVP V_2_ Receptor Antagonists

Several non-peptide AVP V_2_ receptor antagonists have been developed since 1992 [40,41]. These non-peptide AVP antagonists have potent and selective V_2_ receptor antagonists, and can be used orally. Among them, tolvaptan is now being used clinically to treat water retention. Tolvaptan selectively induces free water clearance in normal condition and in the state of impaired water excretion. The aquaretic effect of tolvaptan occurs without inducing natriuresis and kaliuresis or changes in glomerular filtration rate. Namely, tolvaptan has been employed in the treatment for hyponatremia and SIADH in the United States and Europe, and for congestive heart failure and decompensated cirrhosis in Japan. 

Schrier and his associates [42] showed the efficacy of tolvaptan in hyponatremic patients with SIADH, congestive heart failure and liver cirrhosis (SALT-1, SALT-2 studies). Tolvaptan was administered for 30 days in doses of 15, 30 and 60 mg per day in the hyponatremic patients (*n* = 225). Serum Na levels increased by 3.7 ± 2.7 on day 4 and 6.2 ± 4.1 mmol/L on day 30 in the tolvaptan group. The increase was significantly greater than those in the placebo group (0.3 ± 0.1 on day 4 and 1.7 ± 3.6 mmol/L on day 30). In the subsequent SALTWATER study, tolvaptan was administered for four years in 111 patients from the SALT study, and serum Na levels normalized during the four years observation period [43].

The EVEREST and ACTIVE in CHF (chronic heart failure) studies were performed in patients with congestive heart failure [44,45,46]. Konstam *et al.* [45] observed 4133 patients from 359 institutes in the United States and Europe for an average of 9.9 months. Short-term outcome for physical findings of dyspnea, orthopnea, fatigue and edema were significantly improved in patients who received tolvaptan. The percentage improvement was significantly greater than that in the placebo group, and the efficacy appeared less than 12 h after the initial administration of tolvaptan. Tolvaptan promptly increased serum Na levels in the patients, and this increase was maintained for 24 weeks as compared to the placebo group. The diuretic effect of tolvaptan reduced the length of hospital stay from 11.4 to 9.7 days. However, in long-term outcomes tolvaptan treatment did not improve total and cardiovascular mortality in all the patients with heart failure [45]. A subanalysis verified cardiovascular mortality was reduced in a limited number of hyponatremic patients less than 130 mmol/L. In Japan tolvaptan has been widely used in patients with heart failure since 2010. We will be able to report the evidence of clinical efficacy of tolvaptan in heart failure patients in the near future.

## 5. Conclusions

The present review paper demonstrated hyponatremia is associated with heart failure. Impaired renal water excretion is closely associated with an exaggerated release of AVP in heart failure. In the renal collecting duct both short-term and long-term regulation of AQP2 is activated by sustained elevation of AVP, participating in renal water retention and dilutional hyponatremia. In addition, hyponatremia is a predictor for worsening heart failure in patients, and such hyponatremia associated with organ damage predicts the short-term and long-term outcomes of heart failure.
